# Validation of an automated immunoturbidimetric assay for feline serum amyloid A

**DOI:** 10.1186/s12917-022-03456-5

**Published:** 2022-09-28

**Authors:** Elspeth M. Waugh, Hayley Haining, James Harvie, Alison E. Ridyard, P. David Eckersall

**Affiliations:** 1 Veterinary Diagnostic Services, School of Veterinary Medicine, University of Glasgow, Glasgow, UK; 2 Small Animal Clinical Services, School of Veterinary Medicine, University of Glasgow, Glasgow, UK; 3 Institute of Biodiversity, Animal Health and Comparative Medicine, University of Glasgow, Glasgow, UK

**Keywords:** Serum amyloid A, Assay, Validation, Feline, Inflammation, Acute phase protein

## Abstract

**Background:**

Serum Amyloid A (SAA) is a major acute phase protein in cats, increasing rapidly in response to various inflammatory diseases. An automated latex-enhanced immunoturbidimetric assay for human SAA (LZ-SAA, Eiken), previously validated for use in cats, has had further major modification (VET-SAA, Eiken) for specific use in veterinary diagnostic laboratories but has yet to be validated in cats.

**Results:**

Intra-assay and inter-assay CVs for the VET-SAA assay ranged from 1.88–3.57% and 3.98–6.74%, respectively. Linearity under dilution was acceptable with no prozone effect observed. Limit of detection was 1.65 mg/L and limit of quantification was 6 mg/L. Haemoglobin and triglyceride showed no adverse interference, but bilirubin produced positive bias in samples with low SAA. Comparison with the LZ-SAA assay showed significant correlation with proportional bias increasing as SAA concentration increased, likely related to differing calibration standards. SAA was significantly higher in patients with inflammatory disease compared with non-inflammatory disease, and in patients with moderate to highly elevated α1-AGP compared with patients with normal α1-AGP. Improvement of the assay range may be required to fully evaluate differences between disease groups at low SAA levels. Based on ROC curve analysis, at a cut-off point of 20.1 mg/L the VET-SAA assay discriminated between inflammatory and non-inflammatory disease with sensitivity of 0.93 and specificity of 0.99.

**Conclusions:**

The automated VET-SAA assay is a robust, precise, and accurate method for measurement of feline SAA which can clearly identify patients with inflammatory disease. It should be a valuable biomarker for use in feline medicine.

## Background

Serum Amyloid A (SAA) is a small hydrophobic protein with a molecular weight of 9–14 kDa, found in plasma complexed with high-density lipoprotein [1]. It is regarded as a major positive acute phase protein (APP) in most species, including cats, where > 50-fold increases compared with healthy cats have been observed in a variety of inflammatory diseases [2, 3]. In recent years, APPs have increasingly been used as biomarkers of inflammation in veterinary species [4], and a number of studies have demonstrated the utility of SAA for this purpose in cats [2, 3, 5, 6]. This is particularly promising as C-reactive protein (CRP), one of the most frequently measured APPs [7], does not show a significant acute phase response in cats [8].

One barrier to the routine use of SAA as a biomarker is the method of analysis. Many of the reports on feline SAA have employed enzyme linked immunoassay (ELISA) methods, which suffer a number of disadvantages for use in a diagnostic laboratory. These assays are time consuming, requiring one to 2 h of incubation, and the procedure involves several separate steps which contribute to the lower precision of this type of immunoassay format, even if the ELISA assay is performed on robotic instrumentation. The introduction of a commercially available immunoturbidimetric assay for use on automated biochemical analysers has overcome these problems (LZ-SAA, Eiken Chemical Co., Tokyo, Japan). This assay is based on a mixture of anti-human-SAA-specific monoclonal and polyclonal antibodies, which bind to SAA in plasma and produce a change in absorbance. The assay is enhanced by linking the antibodies to latex particles, increasing analytical sensitivity and lowering the limit of detection and quantification. This assay has been validated in cats and performs reliably, discriminating well between healthy cats and those with evidence of inflammation [9].

An improved format of this assay has been developed which uses purely monoclonal antibodies (VET-SAA, Eiken), which should reduce variation between assay batches, and potentially increase specificity [10]. This assay has been validated for use in determination of equine SAA [10] but the use of this test for feline SAA has not been investigated. The objective of this study was to validate the VET-SAA assay for use with feline samples, including assessment of imprecision, accuracy, detection limit, interfering substances, method comparison and overlap performance between groups with different clinical disease status.

## Results

### Assay characteristics

**Fig. 1:**
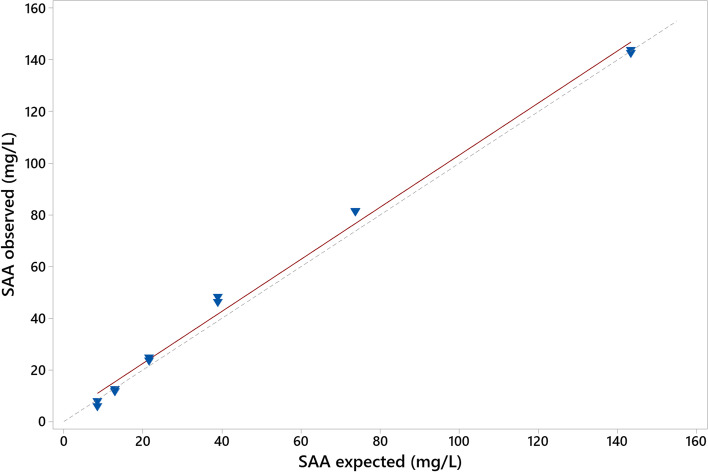
Linearity under dilution of a feline plasma pool with high concentration of SAA. The line of best fit (linear regression) is indicated by the solid line and the line x = y by the dashed line. The regression equation showed no significant deviation of the slope from 1 and the Y-intercept from 0 over a clinically relevant measurand range

Intra-assay and inter-assay CVs ranged from 1.88 to 3.57% and 3.98 to 6.74%, respectively (Table 1). The assay appears linear over the clinically relevant measurand range with no significant deviation of the slope from 1 (1.009, 95% CI 0.949–1.069) or the Y-intercept from 0 (2.26, 95% CI -1.84 - 6.37) on regression analysis (R^2^ = 0.993, Fig. 1).

**Table 1:** Observed intra-assay and inter-assay imprecision

Comparison	No of replicates	Mean (mg/L)	SD	CV (%)
Intra-assay				
Low^a^	11	10.66	0.38	3.57
Mod	21	25.14	0.83	3.30
High	20	115.64	2.17	1.88
Inter-assay				
Low^a^	23	11.72	0.79	6.74
Mod	20	24.47	1.56	6.38
High	20	68.79	2.74	3.98

^a^QC material assayed

The LoB was calculated as 0.44 mg/L (mean_blank_ 0.08, SD_blank_ 0.21) and LoD was 1.65 mg/L (SD_low concentration sample_ 0.73). LoQ was set at 6 mg/L, as this was the lowest level at which TE_obs_ (35.8%) was less than TE_a_ (37.0%).

**Fig. 2:**
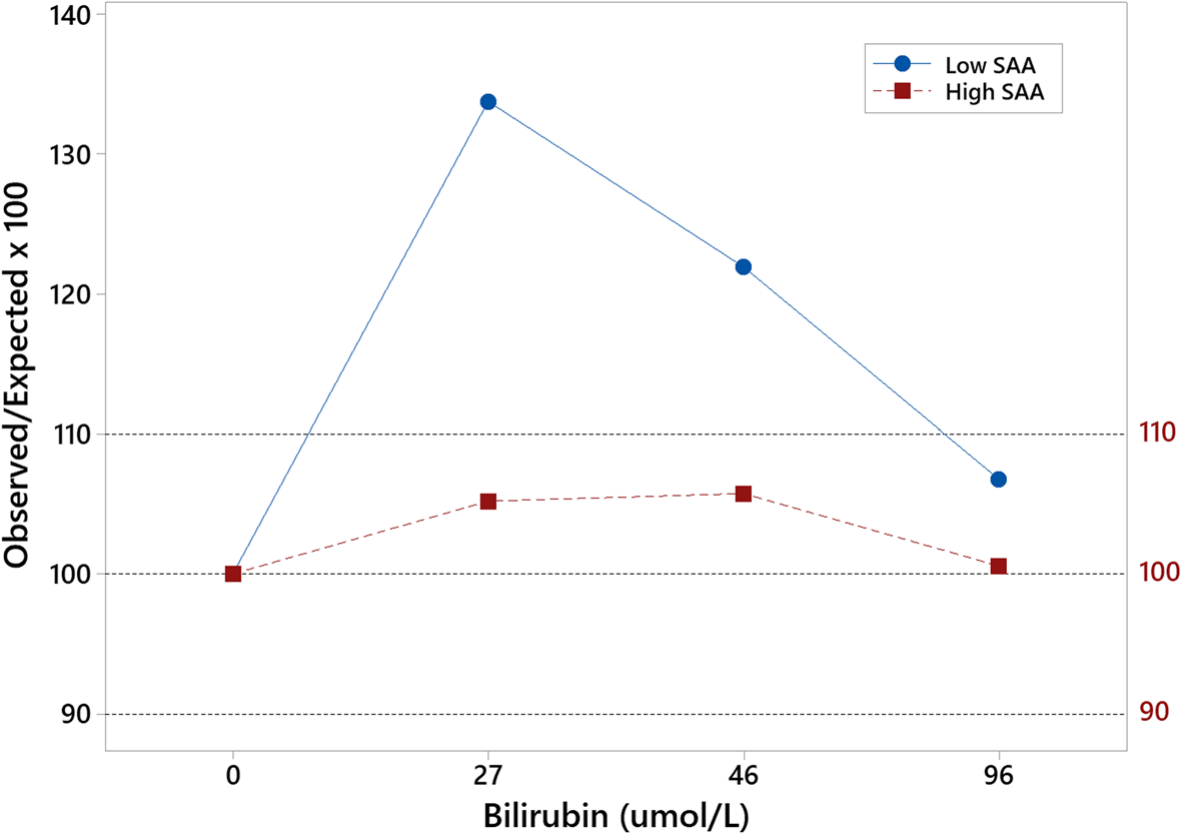
Interferogram for bilirubin demonstrating observed/expected SAA concentration following addition of indicated concentrations of bilirubin to feline samples with low-moderate (blue circles) and moderate-high (red squares) SAA concentrations. Outer dashed horizontal lines indicate level of acceptability (±10% of expected concentration). Bias was within acceptable limits in samples with moderate-high SAA, but bilirubin produced unacceptably high bias when added to samples with a low-moderate SAA concentration

Bilirubin produced a positive bias in samples with low-moderate SAA concentrations (Table 2, Fig. 2). Bias was within acceptable levels for bilirubin in samples with moderate-high SAA and for haemoglobin and triglyceride at both SAA concentrations (Table 2).

**Table 2:** Measured SAA and observed/expected ratios following addition of interfering substances to feline samples with low-moderate and moderate-high SAA concentrations

Interferent	Interferent Concentration	Measured SAA (mg/L) Low-Moderate	O/E (%)^b^	Measured SAA (mg/L) Moderate-High	O/E (%)^b^
Haemoglobin	Blank	18.37	100	49.83	100
	0.146 g/L	18.67	103.02	51.33	103.02
	1.46 g/L	18.28	101.28	50.47	101.28
	14.6 g/L	17.2	96.35	48.01	96.35
Triglyceride	Blank	18.37	100	49.83	100
	2.74 mmol/L	17.95	97.7	53.71	107.81
	4.91 mmol/L	19.44	105.81	51.27	102.91
	9.57 mmol/L	17.4	94.7	51.41	103.19
Bilirubin	Blank	18.37	100	49.83	100
	27 μmol/L	24.57	133.74^a^	52.42	105.2
	46 μmol/L	22.4	121.92^a^	52.69	105.75
	96 μmol/L	19.61	106.77	50.11	100.57

^a^Indicates bias above acceptable limit of ±10% of the blank measurement. ^b^ Observed/Expected (%)

### Method comparison

**Fig. 3:**
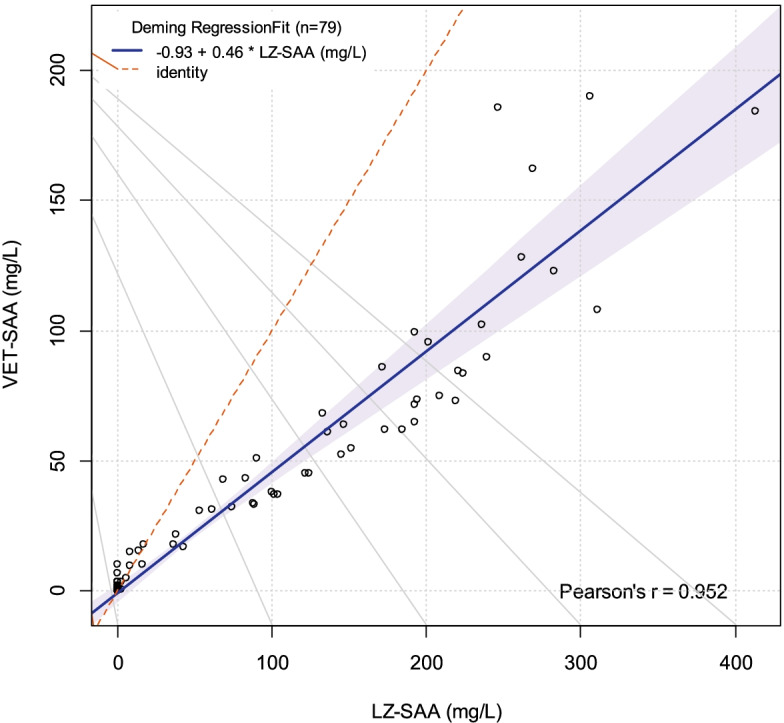
Comparison of SAA concentrations measured using the VET-SAA and LZ-SAA assays. The solid line indicates the line of best fit (Deming regression) with the line of perfect agreement indicated by the dashed line. Results correlated well between the assays, but the regression equation indicated a significant deviation of the slope from 1, consistent with proportional bias

**Fig. 4:**
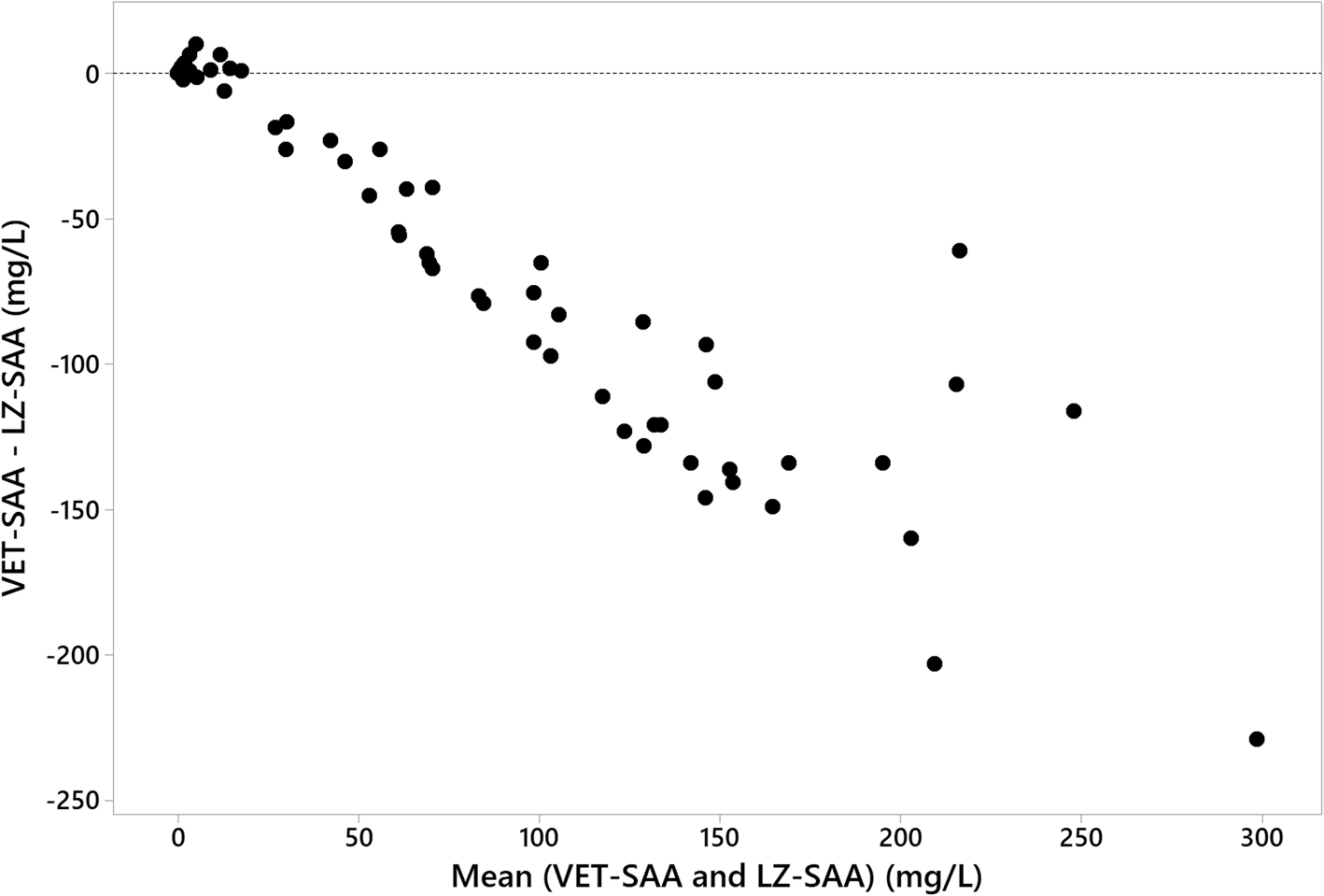
Bland-Altman plot of SAA concentrations measured using the VET-SAA and LZ-SAA assays, which demonstrates negative proportional bias

Results of the VET-SAA assay correlated well with the LZ-SAA assay (R^2^ = 0.95, Fig. 3), with no systematic disagreement (Y-intercept −0.93, 95% CI -4.33 – 2.47). Proportional bias was present, however, as indicated by the slope of the regression equation of 0.46 (95% CI 0.40–0.53). As can be seen on the Bland-Altman plot (Fig. 4), values measured by VET-SAA were lower than those measured by LZ-SAA, with the degree of bias increasing as the SAA concentration increased.

### Overlap performance between patients with different disease status

**Fig. 5:**
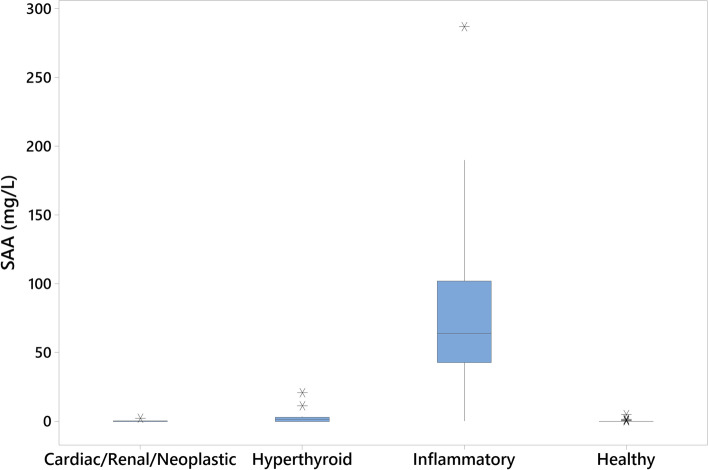
Boxplot of SAA measurements in feline patients grouped by clinical disease status. Boxes indicate interquartile 1–3 range. Outliers are indicated by asterisks. Patients with inflammatory disease had significantly higher SAA levels than those in other groups (*p* < 0.001)

Patients with inflammatory diseases had significantly higher SAA levels than patients in the other groups (*p* < 0.001; Fig. 5, Table 3). Significant differences were also seen between the hyperthyroid and cardiac/renal/neoplastic groups (*p* = 0.002) and the hyperthyroid and healthy groups (*p* = 0.012), however the median SAA levels in all three groups were below the LoQ (Table 3).

**Table 3:** Descriptive statistics for measured SAA in feline patients grouped by clinical disease status

Group	n	Q1 (mg/L)	Median (mg/L)	Q3 (mg/L)	Range (mg/L)
Cardiac/Renal/Neoplastic	13	0.0	0.0	0.6	0.0–2.21
Hyperthyroid	15	0.0	1.57	3.16	0.0–20.9
Inflammatory	27	42.9	64.0	102.0	0.3–287.0
Healthy	54	0.0	0.0	0.13	0.0–4.95

*Q* Quartile

**Fig. 6:**
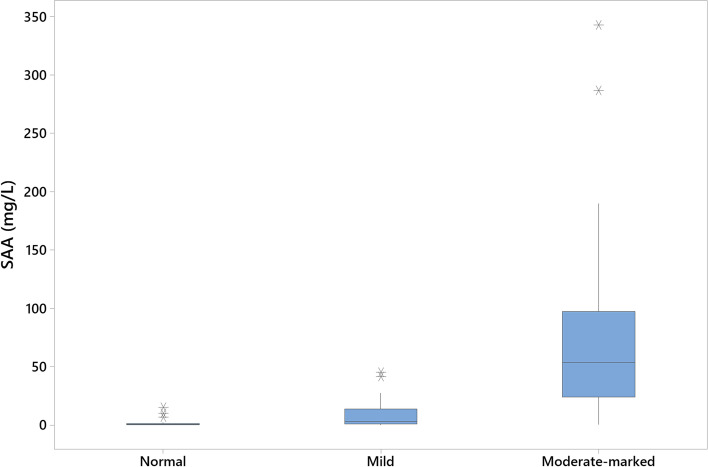
Boxplot of SAA measurements in feline patients grouped by α1-AGP level (normal, mild increase, moderate to marked increase). Boxes indicate interquartile 1–3 range. Outliers are indicated by asterisks. Patients with moderate-markedly increased α1-AGP had significantly higher SAA levels than those in other groups (*p* < 0.001)

In patients grouped by α1-AGP level, those with moderate to markedly increased α1-AGP had significantly higher SAA levels than those in the mildly increased α1-AGP and normal α1-AGP groups (*p* < 0.001; Fig. 6, Table 4). SAA was also significantly higher in patients with mildly increased α1-AGP than those with normal α1-AGP levels (*p* < 0.001), however the median SAA levels were below the LoQ (Table 4).

**Table 4:** Descriptive statistics for measured SAA in feline patients grouped by α1-AGP level

α1-AGP level	n	Q1 (mg/L)	Median (mg/L)	Q3 (mg/L)	Range (mg/L)
Normal	30	0.0	0.53	1.26	0.0–14.96
Mild increase	25	0.96	2.88	13.57	0.0–45.0
Moderate to marked increase	38	23.9	53.6	97.3	0.3–343.0

*Q* Quartile

**Fig. 7:**
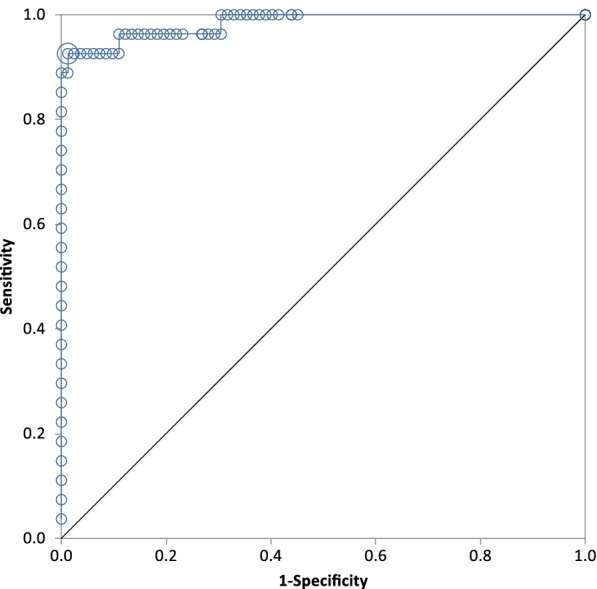
ROC curve for SAA measurements in feline patients designated as having inflammatory or non-inflammatory disease, based on clinical disease status. The non-inflammatory group included healthy patients as well as cardiac, renal, neoplastic and hyperthyroid cases. The AUC was 0.98 with an optimal cut-off point of 20.1 mg/L.

ROC curve analysis was performed to investigate suitable clinical decision levels for patients with inflammatory disease. Patients of known clinical disease status were grouped either as inflammatory disease (*n* = 27) or non-inflammatory (including healthy, cardiac, renal, neoplastic and hyperthyroid cases, *n* = 82). This yielded an AUC of 0.98 (Fig. 7) with an optimal cut-off point of 20.1 mg/L, with associated sensitivity of 0.93 and specificity of 0.99. For comparison, using the LoQ (6 mg/L) as a cut-off point yielded sensitivity of 0.93 and specificity of 0.98.

## Discussion

The VET-SAA assay performed well in validation tests, demonstrating good precision with both intra-assay and inter-assay CVs well below the desirable level of 12.4%. The assay was linear across the clinically relevant analytical range, which is lower than seen with other species [11]. No significant bias was detected on regression analysis and there was no evidence of a prozone effect at the levels tested. There appears to be minimal effect from interfering substances, similar to results seen in other species using this assay [10], although results from interference studies should be interpreted cautiously as the artificial substances added may not mimic exactly the effect of the natural interferent. The one exception was bilirubin in samples with low SAA concentration, which produced unacceptably high positive bias. However, interference from bilirubin was minimal in samples with high SAA concentration, which are likely to be most relevant for clinical diagnosis. The clinical impact of any interference due to icterus may therefore be relatively low, although results with moderate SAA levels should still be interpreted with caution.

Both the established LZ-SAA and the recently introduced VET-SAA assays use the same technology, and so it was expected that results would correlate well. There was, however, significant proportional bias present meaning that the two assays in their current specifications cannot be used interchangeably. One potential source of variation may be the antibodies used in each assay, and the resulting effect on binding of SAA. In horses, it is thought that differences in antibody affinity toward different SAA isoforms may cause variation in results [10], and it could be speculated that a similar effect occurs in feline samples. In this study, however, the proportional bias is attributed to the use of different calibrators, resulting in lower measured SAA levels when using the VET-SAA assay. The use of a WHO-traceable calibrator, as supplied for the VET-SAA assay, is preferred as it improves comparability between assays when calibrated to the same standard [12]. It is possible that had the comparison been performed using the same calibrator for both assays the results would be closer, but this was not possible within this study. For laboratories previously using the LZ-SAA assay to determine SAA in feline serum, it is advised that method comparison is carried out prior to using the VET-SAA assay, with development of new reference intervals and decision levels as the assays are not directly comparable.

As expected, assessment of overlap performance demonstrated significantly higher SAA levels in patients classified as having inflammatory disease, confirming the utility of SAA as a marker of inflammation. Within the non-inflammatory disease groups, hyperthyroid cases had significantly higher SAA, which has been inconsistently reported in the literature [3, 5, 9]. However, the median SAA levels in these groups were below the LoQ and so these findings cannot be regarded as reliable. Improvements in analytical sensitivity and precision at low SAA levels will be required before SAA measurements < 6 mg/L can be confidently evaluated and genuine differences between these groups determined. At the current time, it is advised that results below the LoQ are reported as < 6 mg/L.

When cases with a defined clinical diagnosis are considered, the ROC curve indicates SAA is a highly accurate test for identifying inflammation. At a cut-off of 6 mg/L (the LoQ), both sensitivity and specificity are high. However, this cut-off is the minimum level at which results should be reported, and it may be preferable to use a higher clinical decision limit to ensure high specificity for inflammation. Although not observed in this study, cats with chronic kidney disease have been reported to have increased SAA, albeit at a much lower magnitude than those with inflammatory disease [3, 5, 13]. Increasing the cut-off to 20 mg/L should exclude such cases without a resultant decrease in sensitivity, as most cats with acute inflammation appear to have SAA levels much higher than this (see Fig. 5, Table 3). One hyperthyroid case in this study did have SAA > 20 mg/L, however the most likely explanation for this is undiagnosed (or undisclosed) concurrent inflammatory disease, as suggested by Yuki et al. (2020) [5], rather than an inherent characteristic of hyperthyroidism. Elevated SAA in a patient with non-inflammatory disease should therefore prompt further investigation for inflammation.

Comparison of SAA and α1-AGP levels was consistent with previous work showing a strong correlation between levels of the two acute phase proteins [9], at least for patients with moderately to markedly increased α1-AGP. The significantly higher SAA levels in those with mildly increased α1-AGP compared with the normal α1-AGP group are harder to interpret, since the median level was below the LoQ. However, there were some samples in the ‘mildly elevated’ group which had SAA > 6 mg/L, but below the proposed cut-off of 20 mg/L, raising concern that patients with some evidence of inflammation would be missed using SAA as a single biomarker. One explanation may be related to the temporal differences in kinetics of the two APPs. While both are considered major APPs in the cat, SAA is thought to rise and fall more quickly than α1-AGP [3, 8], and so it is possible that the SAA level in these cats was waning at the time of sampling. This decrease in SAA could signal an improvement in the inflammatory status, suggesting SAA may be helpful in monitoring disease progression and response to treatment, similar to CRP in dogs [7]. One case report has indeed shown the utility of SAA for this purpose in feline pancreatitis [14], although further work will be needed to confirm these findings, and to investigate the SAA response in a wider variety of inflammatory diseases, including localised versus systemic inflammation. Alternatively, the disease could be moving into a more chronic phase of inflammation, where other APPs such as haptoglobin may be more appropriate biomarkers. Taking account of this possibility, recent recommendations advise the use of a panel of APPs including both a major APP, such as SAA in the cat, and a moderate APP, to best evaluate inflammatory status [4].

## Conclusions

The automated VET-SAA assay is a robust, precise and accurate method for measurement of feline SAA. The assay is rapid and readily available, making it suitable for routine diagnostic use. It can clearly identify patients with acute inflammation and has potential utility in both diagnosis and monitoring of inflammatory disease. It should be a valuable biomarker for use in feline medicine.

## Methods

### Sample characteristics

Serum or heparinised plasma samples were initially submitted to the Veterinary Diagnostic Services Laboratories, University of Glasgow, for biochemical analysis. Samples were collected as part of routine diagnostic procedures by the submitting veterinarian. Those with sufficient residual material following completion of all requested tests and either sufficient clinical information to enable diagnosis, and/or concurrent α1-AGP measurements, were selected for SAA analysis (*n* = 123). Additionally, residual samples from 54 clinically healthy cats were obtained from Biobest Laboratories Ltd. (Milton Bridge, UK). Healthy status or clinical diagnosis was determined by the submitting veterinarian, who had no access to results of SAA testing. Both serum and plasma samples were analysed as part of the study, depending on the sample type submitted. Serum and plasma were expected to give equivalent results [15]. Samples were analysed on the day of submission or stored at − 20 °C where this was not possible. Frozen samples were thawed and allowed to come to room temperature prior to analysis.

### SAA assays

The VET-SAA assay (Eiken) was run on an automated analyzer (ABX Pentra 400, Horiba, Grenoble, France) according to the manufacturer’s instructions with 3 μl sample volume. The assay was calibrated using the supplied WHO traceable concentration SAA standard (WHO International Standard 92/680). Control material at two levels (VET-SAA-QC-Low and VET-SAA-QC-High, Eiken) was assayed on each run, prior to sample analysis. Samples with measured SAA above the manufacturer’s stated measurement range (5–200 mg/L) underwent reflex dilution (1:6) and repeat analysis.

For comparison studies, the LZ-SAA assay (Eiken) was used. This assay has been previously validated in cats [9]. It was run on the same analyser according to the manufacturer’s instructions with the same sample volume. The assay was calibrated with the supplied LZ-SAA assay standard which differed from the WHO traceable standard used with the VET-SAA assay.

### Assay validation

To assess assay imprecision, samples from multiple patients were mixed to create sample pools with moderate and high SAA concentrations. Assay imprecision at low SAA concentrations was assessed using QC material (VET-SAA-QC-Low, Eiken). Multiple replicates of sample pools or QC material (see Table 1) were measured either on the same day (intra-assay imprecision), or on separate days (inter-assay imprecision), and mean, SD and CV calculated. Samples for the inter-assay experiments were aliquoted and stored at − 20 °C; aliquots were thawed and allowed to come to room temperature prior to analysis. The limit for desirable imprecision was set at 12.4%, based on data for biological variation of SAA in humans [16], as equivalent data for cats are not currently available.

Linearity and accuracy were assessed by serial 1:2 dilution of a high concentration sample pool (initial concentration 143 mg/L) with a low concentration sample pool (4 mg/L) and measuring each level in duplicate within a single run. Results were plotted against expected values and assessed visually and by linear regression analysis.

Limit of detection was calculated using serial measurements of a blank sample (saline), and a low concentration SAA sample (6 mg/L). These were measured 40 times and 23 times, respectively, over the course of 5 days. Values for mean and SD were calculated and used to calculate the limit of blank (LoB) and limit of detection (LoD) using the following equations [17]:

$$\mathrm{LoB}={\mathrm{mean}}_{\mathrm{blank}}+1.65\left({\mathrm{SD}}_{\mathrm{blank}}\right)$$

$$\mathrm{LoD}=\mathrm{LoB}+1.65\left({\mathrm{SD}}_{\mathrm{low}\ \mathrm{concentration}\ \mathrm{sample}}\right)$$

For limit of quantitation studies, a low concentration sample (11 mg/L) was serially diluted 1:2 with saline and each dilution measured 23 times over 5 days. Limit of quantitation (LoQ) was determined to be the lowest dilution at which total observed error (TE_obs_) was less than the predetermined total allowable error (TE_a_). TE_a_ was set at 37.0%, which reflects data on biological variation of SAA in humans [16]. TE_obs_ for each dilution was calculated using the eq. TE = bias +2SD, where bias was the difference between the measured mean and the expected value (mean of the undiluted sample multiplied by the dilution factor).

The effect of interfering substances was assessed by measuring low-moderate (18 mg/L) and moderate-high (50 mg/L) SAA sample pools which had been spiked with various concentrations of common interferents. To assess the effect of haemolysis, a haemoglobin solution was prepared by adding distilled water to washed red cells. The resulting solution was centrifuged to remove cell debris and the total haemoglobin concentration measured using an Advia 120 Hematology Analyser (Siemens Healthcare Diagnostics Inc., Newark, USA). Dilutions of this solution were added to the SAA sample pools to final concentrations of 0.15, 1.46 and 14.6 g/L. Dilutions of a commercial triglyceride solution (20% Intralipid solution, Sigma-Aldrich, Steinheim, Germany) were added to final concentrations of 2.74, 4.91 and 9.57 mmol/L to mimic lipaemia, and dilutions of a commercial bilirubin solution (Total Bilirubin Calibrator [High], Siemens) were added to final concentrations of 27, 46 and 96 μmol/L to mimic icterus. Levels of triglyceride and bilirubin in the respective preparations were measured on a Dimension Xpand Plus Analyser (Siemens) to confirm concentrations. For each level of interferent, 100 μL of interferent solution was added to 400 μL of sample pool and mixed well. A ‘blank’ preparation consisting of the same volume of saline and sample pool was also made for each SAA level to account for the dilution factor. Each preparation was measured in triplicate in the same assay run. Acceptability was set at +/− 10% of the ‘blank’ measurement [10].

For method comparison experiments 79 samples across the analytical range were tested using both the VET-SAA and LZ-SAA assays, run on the same analyser on the same day. Results were analysed by Deming regression and inspection of a Bland-Altman plot.

To assess overlap performance of the VET-SAA assay between patients with different clinical disease status, patients were assigned to groups based on the clinical information available from the referring veterinarian (Table 5, total samples *n* = 109). Patients with cardiac, renal or neoplastic disease were combined as a single group due to low numbers. Groups were compared using the Kruskall-Wallis test with post-hoc analysis using the Dunn-Bonferroni method. Suitable decision levels were investigated by ROC curve analysis of the different groups.

**Table 5:** Clinical diagnoses for patients grouped by clinical disease status

Group	Diagnoses	n
Cardiac	Hypertrophic cardiomyopathy	1
Healthy	‘Healthy’ samples	54
Hyperthyroid	Hyperthyroidism (*n* = 1 with concurrent diabetes mellitus)	15
Inflammatory	Suspect FIP^a^ (*n* = 24); triaditis, cystitis, orbital abscess (all *n* = 1)	27
Renal	Chronic kidney disease (*n* = 7), protein losing nephropathy (*n* = 1)	8
Neoplastic	Cutaneous haemangiosarcoma, renal lymphoma, salivary adenocarcinoma, soft tissue sarcoma (all *n* = 1)	4

^a^Suspect FIP (feline infectious peritonitis) was assigned based on a combination of clinical information and laboratory data including haematology, plasma proteins, α1-AGP and feline coronavirus antibody levels

Concurrent α1-AGP measurements were available for a subset of samples. To allow comparison of the two acute phase proteins in these patients, results were grouped by α1-AGP level (normal [α1-AGP < 500 μg/mL] *n* = 30, mild increase [α1-AGP 500–1500 μg/mL] *n* = 25, moderate-marked increase [α1-AGP > 1500 μg/mL] *n* = 38). Alpha 1-AGP was measured by ELISA (Avacta Animal Health, Wetherby, UK), and decision limits were set based on the interpretative guidelines in routine use in the laboratory. Groups were compared using the Kruskall-Wallis test with post-hoc analysis using the Dunn-Bonferroni method.

### Statistical software

Statistical analyses were performed using Minitab 19 (Minitab Ltd., Coventry, UK), apart from Deming regression and ROC curve analyses which used StatsDirect v3 (StatsDirect Ltd., Wirral, UK).

## Data Availability

The datasets used and/or analysed during the current study are available from the corresponding author on reasonable request.
